# Revisiting abdominal wall “morbidity” of the extensile anterolateral approach to the thoracolumbar spine

**DOI:** 10.1007/s43390-024-00971-3

**Published:** 2024-09-22

**Authors:** Alekos A. Theologis, Andrew P. Collins, Kanwar Parhar, Munish C. Gupta

**Affiliations:** 1 Department of Orthopaedic Surgery, University of California - San Francisco (UCSF), 500 Parnassus Ave, MUW 3rd Floor, San Francisco, CA 94143 USA; 2 Department of Orthopaedics and Sports Medicine, University of Washington, Seattle, WA USA; 3 Elson S. Floyd College of Medicine, Washington State University, Pullman, WA USA; 4 Department of Orthopaedic Surgery, Washington University in St. Louis, St. Louis, MO USA

**Keywords:** Lumbar spine, Anterior lumbar interbody fusion, Anterolateral approach, Retroperitoneal, Patient-reported outcome measures, Morbidity

## Abstract

**Purpose:**

To assess patients’ perceptions of their abdominal wall following extensile anterolateral approaches to the thoracolumbar spine for adult spinal deformity (ASD) using validated questionnaires.

**Methods:**

Adults who underwent anterior–posterior thoracolumbar spinal operations to the pelvis for ASD in which the anterior fusion was performed through an extensile anterolateral approach were reviewed. Three questionnaires were administered at least 1 year following surgery and included The Abdominal Core Health Quality Collaborative Survey (AHS-QC), The Patient Scar Assessment Scale (PSAS), and The Anterior Abdominal Incision Questionnaire (AAIQ).

**Results:**

Fifty-one patients (80.4% female, median age 65 years) were included. Average follow-up was 2.8 ± 1.7 years. Average number of anterior fusion levels was 3.5 ± 1.4. Patients achieved high satisfaction rates from surgery (74.5%). AAIQ responses included postoperative pain (33.3%), bulging (41.7%), and limitations in daily activities (18.8%) with only 15.7% experienced moderate–severe pain related to their incisions and only 6.3% seeking treatment for their scars. Post-operatively, 63.2% had a neutral or improved self-image of their torso and trunk, while only 10.2% stating it was much worse. Patients’ overall opinion of their scar compared to their normal skin was very positive [average 2.75 ± 2.93 (10 = worst possible scar)]. Favorable scores were also reported for color difference, stiffness, change in thickness, and irregularity in their abdominal scar compared to normal skin.

**Conclusions:**

Following extensile anterolateral approaches to the thoracolumbar spine for ASD, the majority of patients reported mild pain, mild functional limitations, good cosmesis, and high satisfaction rates with their anterior incisions based on validated questionnaires.

## Introduction

Multi-level interbody support of the lumbar spine can be accomplished through a variety of surgical exposures. The open anterolateral approach was traditionally utilized for its ease of access, direct visualization from T12 to S1, and the ability to release the anterior longitudinal ligament (ALL) at each level. While this provided an excellent ability to restore lumbar lordosis, achieve fusion, and facilitate sagittal and coronal deformity correction, the extensile nature of the incision and subsequent concerns for abdominal wall cosmesis and hernias were deemed “morbid”. While the perception of the approach’s “morbidity” were primarily anecdotal, *Kim *et al*.* reported this approach had an appreciably high rate of postoperative pain, bulging, and functional disturbance in adult spinal deformity (ASD) patients [1]. The authors concluded that surgeons should use caution when recommending this approach to future ASD [1]. However, the study utilized a non-validated questionnaire, thus jeopardizing the validity of the results. Nevertheless, the results of the study confirmed surgeons’ anecdotal concerns about this approach and resulted in the development of alternative and less invasive surgical approaches to achieve lumbar interbody support, including the direct lateral interbody (DLIF) approach and the anterior to psoas interbody approach (ATP).

The utilization of DLIF and ATP have flourished over the last 10–15 years as a result of their minimally invasive nature [2]. As their incisions are smaller and the abdominal wall musculature is split, rather than divided, they have a lower rate of incisional hernias and are cosmetically more appealing compared to the extensile anterolateral approach [3, 4]. However, DLIF and ATP have unique and important limitations and complication profiles [5]. As such, these limitations and potentially devastating complications bring into question whether smaller incisions and abdominal wall preservation are justified. It also demands that the “morbidity” of the traditional extensile anterolateral approach to the lumbar spine be re-examined.

In this study, we propose to comprehensively assess patients’ perceptions of their abdominal wall following ALIFs in the setting of ASD operations performed through the extensile anterolateral approach to the lumbar spine. We use validated questionnaires for wound cosmesis and abdominal wall function and associated disability with the goal to more comprehensively and accurately evaluate the “morbidity” of this approach.

## Materials and methods

### Patient population

The Institutional Review Board approved all aspects of the current study. All participants provided written informed consent prior to the commencement of the study. Participants eligible for this study were identified with a retrospective review of a prospective single surgeon database of spinal operations performed at a single academic medical institution. Inclusion criteria consisted of adult patients (> 18 years) who had undergone elective, multi-level ALIF operations via an extensile anterolateral approach with a minimum of 1-year follow-up on each of the 3 patient questionnaires. The same single vascular surgeon and his team performed each approach completely and also performed each abdominal wall closure on every case. All anterior operations were performed through an extensile anterolateral approach—no patients had this approach combined with another minimally invasive approach to the anterior thoracolumbar spine [i.e., no direct lateral/transpsoas approach (i.e., DLIF) or minimally invasive anterior to psoas approach (i.e., ATP)]. All anterior operations involved complete/radical discectomies with release of the ALL and fusion with morselized bone graft without structural interbody support for levels cranial to L4 and with structural interbody support (femoral ring allografts) at the L4–5 and L5–S1 levels. At L4–5 and L5–S1, 6.5 mm cancellous screws through a washer were placed on one endplate to serve as a buttress to prevent anterior graft dislodgement. No segmental anterior instrumentation (i.e., rods/screws crossing a disc space) were performed. All posterior instrumentation constructs consisted of polyaxial pedicle screws for thoracic levels and iliac fixation, while fixed/posted pedicle screws were used in the lumbar spine. Patients with a prior abdominal surgery, surgery using less invasive approaches (i.e., ATP or DLIF), or those undergoing spinal surgery via a posterior-only approach or for traumatic, malignant, and/or infectious etiologies were excluded. While the less invasive lateral and anterior lumbar interbody fusion approaches are viable techniques to address ASD, the extensile approach was used given it is the preferred and standard practice of the senior surgeon of this article to address spinal deformity.

### Data collection and questionnaires

Patient and perioperative characteristics, including age, sex, laterality of incision, length of anterior fusion construct, specific levels fused, and length of postoperative follow-up, were collected. The following 3 questionnaires were administered to patients in the clinic: the Abdominal Core Health Quality Collaborative Survey (AHS-QC), Patient Scar Assessment Scale (PSAS), and the Anterior Abdominal Incision Questionnaire (AAIQ). A “Do not wish to answer” option was also included in each survey. During these evaluations, radiographs were not obtained for all patients given their inclusion was not approved by the IRB, as many evaluations were made upon request and were not part of routine postoperative follow-up.

The AHS-QC is recognized as a validated survey assessing abdominal wall integrity and core stability [6]. In this study, patients rated each question regarding their abdominal wall on a descriptive scale with 6 options ranging from “Strongly Agree” to “Strongly Disagree”. This survey investigated pain at the incision site along with function in overall health, moderate and strenuous activities, walking, sexual function, activities of daily living, productivity, and mood.

The PSAS is a validated tool to assess scars in a suitable, reliable, and complete evaluation by focusing on pain, itching, stiffness, thickness, and irregularity of the scar compared with normal skin [7]. It is commonly employed to evaluate scars following plastic surgery operations. The survey consists of 7 questions regarding a patient’s scar, and each is rated on a scale of 1 to 10. The scores for the first 6 questions are summed and a total score, "The PSAS Score” is reported for each patient. A score of 0 translates to the scar being no different than normal skin and a score of 60 translates to the worst scar possible.

The AAIQ lacks prior validation, though it was utilized to present a novel method assessing scar characteristics, pain, and patient perspectives on their incision [1]. This survey consists of 10 varying questions about the patient’s anterior abdominal wall incision, examining pain, appearance, bulging, daily life, and the patient’s opinion of the surgery. To assess the degree of pain and perception of appearance, the Visual Analog Scale was used.

### Statistical analysis

Data analysis was conducted with IBM SPSS (IBM Corp. Released 2021. IBM SPSS Statistics for Macintosh, Version 28.0. Armonk, NY: IBM Corp). Descriptive statistics were conducted for patient characteristics, perioperative variables, and a summary of survey questions. Univariate analyses were used to determine the effect of patient characteristics and perioperative data on the outcomes of the ACH-CS and PSAS questionnaires. For independent variables with a *p*-value < 0.1, a multivariate analysis was to be conducted, though no variables met this threshold. Patients who stated they did not wish to answer specific questions had their individual answers omitted from analyses and summed total outcomes scores removed.

## Results

### Descriptive analysis (Table 1)

**Table 1:** Characteristics of study population

Variable	*n* ± SD (%)	ACH-QC total score univariate analysis	PSAS total score univariate analysis
Age	62.9 ± 12.1	*p* = 0.442	*p* = 0.070
Gender		*p* = 0.378	*p* = 0.160
Male	10 (19.6)		
Female	41 (80.4)		
Average follow-up	2.8 ± 1.7 years		
Laterality of approach		*p* = 0.864	*p* = 0.778
Left	49 (96.1)		
Right	2 (3.9)		
Length of fusion	3.5 ± 1.4	*p* = 0.699	*p* = 0.805
2	13 (25.5)		
3	15 (29.4)		
4	10 (19.6)		
5	9 (17.6)		
6	3 (5.9)		
7	1 (2.0)		
Levels fused			
T9–T10	1		
T10–T11	2		
T11–T12	6		
T12–L1	13		
L1–L2	18		
L2–L3	24		
L3–L4	39		
L4–L5	46		
L5–S1	30		
Fusion levels			
T9–L4	1		
T10–L4	1		
T11–L4	2		
T11–L5	2		
T12–L4	1		
T12–L5	6		
L1–L5	4		
L1–S1	1		
L2–L5	1		
L2–S1	5		
L3–L5	1		
L3–S1	14		
L4–S1	12		

A total of 51 patients with > 1 year follow-up were included for analysis (69 patients were excluded for having surgery within the year that this study started and therefore did not have 1 year of follow-up). All patients who were successfully contacted participated in the study and completed the questionnaires completely. The median participant age was 65 years (IQR 57–72) and 80.4% were female. The average follow-up duration was 2.8 ± 1.7 years. The vast majority (96.1%) of exposures/incisions were performed from the left. The average length of anterior lumbar fusion spanned 3.5 vertebrae (range, 2–7), with the most cranial level at T9 and the most caudal level at the sacrum. The most commonly fused vertebrae included L4–5 (*n* = 46, 90.2%), L3–4 (*n* = 39, 76.5%), and L5–S1 (*n* = 30, 58.8%). There were no vascular injuries or dural openings during the anterior operations. Additionally, no wound infections developed associated with the anterolateral approaches. On univariate analysis, age, gender, incisional laterality, and length of anterior fusion did not significantly influence AHS-QC or PSAS total scores.

### AHS-QC

Results of the validated AHS-QC questionnaire are presented in Table 2. On average, patients reported their recent pain to be none or mild at its worst (*n* = 31, 60.8%) and at baseline levels (*n* = 36, 70.6). The majority of the participants reported no impact on their activities of daily living (*n* = 36, 64.2%), moderate activity (*n* = 32, 61.5%), strenuous tasks (*n* = 32, 61.5%), walking (*n* = 37, 71.2%), and sexual activity (*n* = 32, 65.3%) secondary to their abdominal wall. Patients most often strongly disagreed that their abdominal wall caused them to stay at home (*n* = 35, 70.0%) and accomplish less at work (*n* = 32, 66.7%). Fewer than a third of patients stated they would strongly agree that their abdominal wall had a huge impact on their health (*n* = 14, 28.0%).

**Table 2:** Abdominal Core Health Quality Collaborative (ACH-QC) survey detailed results

Abdominal core health quality collaborative survey	Response	*n* (%)
In the past 7 days how intense was your pain at its worst?	No pain	23 (45.1)
	Mild pain	8 (15.7)
	Moderate pain	13 (25.5)
	Severe pain	4 (7.8)
	Very severe pain	3 (5.9)
In the past 7 days how intense was your average pain?	No pain	25 (49.0)
	Mild pain	11 (21.6)
	Moderate pain	9 (17.6)
	Severe pain	5 (9.8)
	Very severe pain	1 (2.0)
What is your pain right now?	No pain	27 (52.9)
	Mild pain	13 (25.5)
	Moderate pain	7 (13.7)
	Severe pain	3 (5.9)
	Very severe pain	1 (2.0)
My abdominal wall has a huge impact on my health	Strongly disagree	17 (34.0)
	Moderately disagree	5 (10.0)
	Slightly disagree	1 (2.0)
	Slightly agree	6 (12.0)
	Moderately agree	7 (14.0)
	Strongly agree	14 (28.0)
My abdominal wall causes me physical pain	Strongly disagree	27 (54.0)
	Moderately disagree	0 (0.0)
	Slightly disagree	0 (0.0)
	Slightly agree	8 (16.0)
	Moderately agree	9 (18.0)
	Strongly agree	6 (12.0)
My abdominal wall interferes when I perform strenuous activities (heavy lifting)	Strongly disagree	23 (48.9)
	Moderately disagree	3 (6.4)
	Slightly disagree	2 (4.3)
	Slightly agree	4 (8.5)
	Moderately agree	6 (12.8)
	Strongly agree	9 (19.1)
My abdominal wall interferes when I perform moderate activities (bowling, bending over)	Strongly disagree	24 (51.1)
	Moderately disagree	3 (6.4)
	Slightly disagree	2 (4.3)
	Slightly agree	2 (4.3)
	Moderately agree	8 (17.0)
	Strongly agree	8 (17.0)
My abdominal wall interferes when I walk or climb stairs	Strongly disagree	25 (53.2)
	Moderately disagree	6 (12.8)
	Slightly disagree	2 (4.3)
	Slightly agree	5 (10.6)
	Moderately agree	4 (8.5)
	Strongly agree	5 (10.6)
My abdominal wall interferes when I dress myself, take showers, or cook	Strongly disagree	27 (55.1)
	Moderately disagree	3 (6.1)
	Slightly disagree	2 (4.1)
	Slightly agree	7 (14.3)
	Moderately agree	6 (12.2)
	Strongly agree	4 (8.2)
My abdominal wall interferes with my sexual activity	Strongly disagree	23 (51.1)
	Moderately disagree	4 (8.9)
	Slightly disagree	2 (4.4)
	Slightly agree	3 (6.7)
	Moderately agree	5 (11.1)
	Strongly agree	8 (17.8)
I often stay home because of my abdominal wall	Strongly disagree	35 (70.0)
	Moderately disagree	6 (12.0)
	Slightly disagree	3 (6.0)
	Slightly agree	0 (0.0)
	Moderately agree	2 (4.0)
	Strongly agree	4 (8.0)
I accomplish less at home because of my abdominal wall	Strongly disagree	30 (60.0)
	Moderately disagree	6 (12.0)
	Slightly disagree	2 (4.0)
	Slightly agree	4 (8.0)
	Moderately agree	2 (4.0)
	Strongly agree	6 (12.0)
I accomplish less at work because of my abdominal wall	Strongly disagree	32 (66.7)
	Moderately disagree	3 (6.3)
	Slightly disagree	2 (4.2)
	Slightly agree	4 (8.3)
	Moderately agree	4 (8.3)
	Strongly agree	3 (6.3)
My abdominal wall effects how I feel every day	Strongly disagree	27 (54.0)
	Moderately disagree	6 (12.0)
	Slightly disagree	1 (2.0)
	Slightly agree	4 (8.0)
	Moderately agree	3 (6.0)
	Strongly agree	9 (18.0)
I often feel blue because of my abdominal wall	Strongly disagree	31 (60.8)
	Moderately disagree	5 (9.8)
	Slightly disagree	0 (0.0)
	Slightly agree	4 (7.8)
	Moderately agree	5 (9.8)
	Strongly agree	6 (11.8)

### PSAS

Results of the validated PSAS questionnaire are presented in Table 3. The average total PSAS score was 10.77 ± 11.41 out of a total of 60 (a score of 0 translates to the scar being no different than normal skin and a score of 60 translates to the worst scar possible). On average, patients were least likely to experience itching and pain from their abdominal scars with 11.7% (*n* = 6) and 3.9% (*n* = 2) reporting moderate-to-severe pain or itching, respectively. Patients reported relatively higher scores (albeit still low) for irregularity (2.71 ± 3.32 out of 10) and difference (2.54 ± 3.03 out of 10) from their non-scarred skin. The patients’ average overall opinion of their scar compared to their normal skin was very favorable (2.75 ± 2.93 out of 10).

**Table 3:** Patient Scar Assessment Scale (PSAS) survey results

Questions	Average ± SD
1) Has the scar on the front/side of your abdomen been painful the past few weeks?*	0.84 ± 1.78
2) Has the scar on the front/side of your abdomen been itching in the past few weeks?*	0.43 ± 1.06
3) Is the color of the scar on the front/side of your abdomen different from the color of your normal skin at present?*	2.24 ± 2.86
4) Is the stiffness of the scar on the front/side of your abdomen different from your normal skin at present?*	2.08 ± 2.97
5) Is the thickness of the scar on the front/side of your abdomen different from your normal skin at present?*	2.54 ± 3.03
6) Is the scar on the front/side of your abdomen more irregular than your normal skin at present?*	2.71 ± 3.32
7) What is your overall opinion of the scar on the front/side of your abdomen compare to your normal skin?*	2.75 ± 2.93
Total PSAS Score (0 = best; 60 = worst)	10.77 ± 11.41

^*^ 0 = no, not at all; 10 = yes, very much

### AAIQ

Results from the non-validated AAIQ questionnaire are presented in Table 4. Sixteen patients (33.3%) reported that they experienced pain and 20 patients (41.7%) experienced skin bulging around the area of their anterior abdominal scar. Twelve patients (23.5%) complained of both of these symptoms. Additionally, only 18.8% (*n* = 9) stated the scar limited their ability to fulfill tasks around their home (Table 4). Thirty patients (58.8%) had no complaints about their incision/approach based on the AAIQ questions. On average, patients experienced a pain score of 1.35 ± 2.18 out of 10 from their scar. Only 3 patients (6.3%) sought treatment for their scars. Cosmetically, 63.2% (*n* = 31) of patients had neutral or positive opinions on the look of their torso post-operatively. The average patient rating for their anterior incision was 6.18 ± 3.46 (10 being the same as normal skin). While 74.5% (*n* = 38) of patients reported probably or definitely repeating the surgery, 70.5% (*n* = 36) stated a preference to have the surgery performed through a posterior-only approach if the outcomes could be the same.

**Table 4:** Anterior Abdominal Incision Questionnaire detailed results

Anterior abdominal incision questionnaire	Response	*n* (%)
1) Do you have pain in the area of your anterior (front or side) incision?	Yes	16 (33.3)
2) Do you have skin bulging around or over your anterior (front or side) scar?	Yes	20 (41.7)
3) Have you had any treatment for, or to, your anterior (front or side) incision?	Yes	3 (6.3)
4) Does your anterior (front or side) incision on your abdominal area limit your ability to do things around the house?	Yes	9 (18.8)
5) If you had the surgery to do over again, would you have the same treatment of your spine?	Definitely yes	26 (51.0)
	Probably yes	12 (23.5)
	Not	10 (19.6)
	Probably no	1 (2.0)
	Definitely no	2 (3.9)
6) If you could have the surgery all over again (with the same results), and it could be done all from the back (no front or side incision), would you have the surgery?	Definitely yes	27 (52.9)
	Probably yes	9 (17.6)
	Not sure	13 (25.5)
	Probably no	1 (2.0)
	Definitely no	1 (2.0)
7) Compared with before treatment, how do you feel your trunk and torso look now?	Much better	12 (24.5)
	Better	12 (24.5)
	Same	7 (14.3)
	Worse	13 (26.5)
	Much worse	5 (10.2)
8) How often do you have pain of your anterior (front or side) incision?	Rarely	36 (70.6)
	Occasionally	7 (13.7)
	Frequently	4 (7.8)
	Everyday	4 (7.8)
9) What is the level of pain in the area of your anterior (front or side) incision from 0 to 10?	1.35 ± 2.18	–
10) Please rate the appearance of your anterior (front or side) incision on a scale from 0 to 10	6.18 ± 3.46	–

## Discussion

In recent years, patient-reported questionnaires and outcomes have become the gold standard for the evaluation of symptoms and function to derive operative success in adult deformity surgery [6–9]. While few studies have directly assessed complications concerning approach-specific problems, *Kim *et al*.* reported that patients undergoing the open anterolateral approach had high rates of postoperative pain, bulging, and functional disturbance of the abdominal wall [1]. The authors concluded that surgeons should use caution when recommending the approach to future patients, confirming surgeons’ anecdotal concerns about the approach. However, the authors utilized a non-validated questionnaire, compelling further investigation into the true “morbidity” associated with the extensile anterolateral approach. In the current study, we utilized the same AAIQ as *Kim *et al*.* [1] as well as two additional validated questionnaires (AHS-QC and PSAS) to develop a more comprehensive understanding of patients’ perspectives on wound cosmesis, abdominal wall function, and associated disability.

### Pain and functional disturbance

In our series, as noted in the AAIQ, while the minority of patients reported either pain (33.3%), bulging (41.7%), or limitations in their daily activities (18.8%) associated with their anterior incision, there was some overlap in symptoms. Patients enrolled in the study by *Kim *et al*.* reported very similar rates of pain (32.3%), bulging (43.5%), and hindrance to activities of daily living (24.2%) [61.3% of the cohort expressed at least one of these complaints] attributable to the abdominal incision [1]. Given these similarities and the fact that the study by *Kim *et al*.* [1] reported these findings at a minimum of 5-year follow-up suggest that pain and functional disturbance related to the anterior incision may not change considerably after 1 year of follow-up. While our study demonstrated a high incidence of anterior incision-related pain on the AAIQ, the average pain score was very mild (1.35 ± 2.18) and only 15.7% of patients reported moderate-to-severe pain (on the visual analog scale) [10]. This is consistent with prior studies [11, 12]. For example, *Horton *et al*.* demonstrated that 15.7% of adults experienced moderate-to-severe pain following the thoracolumbar approach. [13].

While the AHS-QC survey has been a validated tool in assessing abdominal core health following several abdominal surgeries, the survey has not been previously used to assess the anterolateral approach to the lumbar spine [14]. Patients in our cohort reported an average total score of 34.88 ± 21.69, citing the lowest scores and least impact for current pain level and interference of daily activities. While relatively higher scores were reported for abdominal wall impact on overall health and interference with moderate and strenuous activities, the scores were still low and were in the minority of the cohort.

The PSAS is another validated tool for assessing the tactile characteristics and morbidity caused by surgical scars. Patients in our study reported minimal recent average pain (0.84 ± 1.78) and itching (0.43 ± 1.06) caused by their scars. Only 11.8% reported moderate pain and itching and 3.9% reported severe pain or itching at their most recent follow-up. These data are consistent with postoperative surgical scar pain with the other surveys utilized in this study.

Accessing a wider approach and more cranial portions of the rib heads in thoracolumbar deformity surgery may increase the risk of intercostal nerve irritation, influencing the formation of pain, bulging, and sensory disturbances along an anterior incision [15]. In the current study, neither the AHS-QC nor the PSAS were influenced by the length of anterior fusion, level of anterior spinal fusion, and/or or laterality of the anterior approach on univariate analysis. Age and sex also did not significantly affect the postoperative incisional outcomes on univariate analysis.

### Cosmesis and satisfaction

The majority of patients reported minimal negative impact on their cosmesis and self-image related to their anterior abdominal scar. The average rating for their abdominal incisional area on the AAIQ was 6.18 ± 3.46 (range, 1–10) and 63.3% of patients had a neutral or improved self-image of their torso and trunk. While 36.7% of patients reported worse self-image only 10.2% stated that it was much worse. These findings are similar to those of the cohort in the study by *Kim *et al. [1] The AHS-QC also revealed that most patients did not feel blue (69.6%) or affect how they felt every day (66.1%) because of their abdominal wall. These findings raise the important question of whether it is acceptable for approximately one third of patients to feel uncomfortable or unhappy with their cosmesis. The answer to this will likely vary from reader to reader who will have different thresholds for what is acceptable and what is not. Other factors that may inform the readers’ opinion are answers to other questions in the questionnaires, particularly in PSAS. For example, the PSAS demonstrated that patients’ overall opinion of their scar compared to their normal skin was very favorable (average score 2.75 ± 2.93). Furthermore, generally approving opinions are highlighted by the fact that only 6.3% of patients sought treatment for concerns (i.e., symptoms and/or appearance) with their abdominal incision. Patients also reported favorable scores, on average, for color differences, stiffness, change in thickness, and irregularity in their scar compared to normal skin on the PSAS. While the validated surveys utilized in this study assessed self-image directly related to their surgical scars, the AAIQ evaluated self-image in relation to their trunk and torso, which may have been impacted by weight gain or loss, change in their spinal deformity, and/or other changes that may have occurred following the anterior and/or posterior spinal operations; self-image may be impacted by an array of reasons.

Patients’ general opinions and satisfaction with their surgery were assessed in the AAIQ. Importantly, 74.5% of patients reported they would have the same surgical treatment for their spine if they could do it over again, demonstrating a high level of patient satisfaction following open anterolateral lumbar fusion. However, if the same functional results could be guaranteed with a posterior-only approach, 70.5% of patients stated they would prefer this. While this answer may be taken to imply patients are dissatisfied with the “morbidity” of the anterolateral approach, that presumption would be inaccurate, as the question does not query the reasons for the patients’ preference for a posterior-only approach. It may be that patients are satisfied with the anterior abdominal incision (associated pain, appearance, etc.), but were less enthusiastic about other factors related to the ALIF being performed, including added operative time, two anesthetics and extended length of hospital stay if the two approaches were staged on different days. Nevertheless, the responses to this question and the same one in the study by *Kim *et al*.* [1] facilitated a trend toward posterior-only approaches and less invasive anterolateral approaches (i.e., DLIF and ATP) in the treatment of adult spinal deformity.

As DLIF and ATP utilize smaller incisions and less invasive access to the lumbar spine, lower rates of incisional hernias and more cosmetically appealing scars have been focal points of each compared to the extensile anterolateral approach. However, these approaches have distinct limitations and complication profiles that may reduce their ability to restore sagittal alignment safely and effectively in the lumbar spine if the ALL is not released [4, 16–18]. For example, the DLIF struggles to access L4–L5 and L5–S1 and is associated with higher rates of postoperative anterior thigh numbness and quadriceps palsy [16]. While the ATP approach can access lumbar levels from L1–S1, the limited anatomic visualization risks vascular and ureteral injuries [4]. The release of the ALL via mini-open DLIF and ATP approaches may not be conducted due to difficulty and risk of injury to the major vessels, limiting the ability of these approaches to restore lordosis and facilitate deformity correction. Releasing and gaining adequate correction of the lumbosacral junction is paramount to increase the likelihood of getting a fusion. Achieving low lumbar lordosis has been reported to be greater with ALIF rather than posterior approaches such as TLIF and PLIF [19, 20]. Similarly, fractional lumbosacral curves are easier to release with an ALIF. As such, these limitations and potentially devastating complications bring into question whether smaller incisions and abdominal wall preservation are justified.

### Limitations

The results of our study should be considered in the context of its limitations. Its retrospective design increases the risk of selection bias in our cohort. The cohort of 51 patients is limited in size, but comparable to a similar study performed by *Kim *et al*.* [1]. While there was heterogeneity in regard to follow-up and levels of ALIF performed, thus varying location of incision on the anterolateral wall, univariate analysis found time to follow-up and levels and length of the anterior fusions were not associated with responses to the AHS-QC and PSAS. Despite this variability, all procedures were performed by the same vascular surgeon and a single spine surgeon at one medical center. While this may control for differences in operative technique and confounding, it may reduce the external validity of our findings. The results were also not compared to more minimally invasive approaches (i.e., DLIF and ATP) to the lumbar spine, which are proven effective strategies to correct ASD and also hold the benefits of involving smaller incisions and do not require coordination with an approach surgeon. Furthermore, outcomes and “morbidity” are influenced by a variety of patient factors (i.e., employment status, medical comorbidities) and pre- and postoperative factors, which were not queried in this study. Radiographic spinal alignment parameters and assessment of fusion via CT scans were also beyond the scope of this study, as neither were obtained at the time of questionnaire completion. We are not clear as to whether functional and radiologic outcomes affected how patients answered the questionnaires in this study. Nevertheless, this topic may benefit from a multi-center analysis comparing outcomes and patients’ opinions from multiple surgeons and different surgical approaches as well as additional perioperative surgical parameters, radiographic alignment parameters, and patient-reported outcome measures. Another limitation is that the surveys were administered at one time-point in follow-up, which precludes assessment of changes in patient perceptions of their anterior scars over time. Additionally, the patient questionnaires utilized were either non-validated (AAIQ) or have not been studied in patients undergoing anterolateral spinal fusion (AHS-QC and PSAS). In this study, validation of the AAIQ was beyond its scope. Noteworthy is that the AHS-QC questionnaire has two non-specific questions about pain, which renders it less reliable as an anatomically focused tool for assessing abdominal wall-related outcomes. Lastly, patient questionnaires may be at risk of observer bias as healthier patients are less inclined to return for follow-up, and patients potentially more bothered by their incisions are more likely to take the time to complete the surveys. While our aims were to determine patients’ perspective on their abdominal surgical scars, an objective assessment of their scars with measurements of size, bulging, and color would have strengthened our findings [21–23]. Despite these limitations, our results add a deeper understanding of and shed greater light on patients’ perceptions and satisfaction with extensile open anterolateral approaches to the lumbar spine to address adult spinal deformity. Our study will ideally stimulate further discussion around this topic and facilitate other similar investigations focused on the extensile open anterolateral approach and minimally invasive multi-level lateral surgeries.

## Conclusions

This is the first study utilizing validated questionnaires to assess surgical scar morbidity in adults undergoing extensile open anterolateral approaches and thoracolumbar fusion for adult spinal deformity. While patients reported appreciable rates of postoperative pain, bulging, and limitations in daily activities based on a non-validated questionnaire, only 15.7% reported moderate–severe pain. Scar cosmesis and abdominal wall function were deemed highly favorable based on two validated questionnaires. Furthermore, 74.5% of patients were satisfied with their surgical outcomes and reported they would undergo the same surgery again given their current function. As the responses are not as damning as previously reported, our results may be valuable in shared decision-making processes with adult patients considering surgery for thoracolumbar spinal deformity.

## Data Availability

The data that support the findings of this study are available from the corresponding author, [AAT], upon reasonable request.
